# Interaction with adipocyte stromal cells induces breast cancer malignancy via S100A7 upregulation in breast cancer microenvironment

**DOI:** 10.1186/s13058-017-0863-0

**Published:** 2017-06-19

**Authors:** Minako Sakurai, Yasuhiro Miki, Kiyoshi Takagi, Takashi Suzuki, Takanori Ishida, Noriaki Ohuchi, Hironobu Sasano

**Affiliations:** 1 Department of Pathology, Tohoku University Graduate School of Medicine, 2-1 Seiryo-machi, Aoba-ku, Sendai, Miyagi 980-8578 Japan; 2 Department of Disaster Obstetrics and Gynecology, International Research Institute of Disaster Science, Tohoku University Graduate School of Medicine, 2-1 Seiryo-machi, Aoba-ku, Sendai, Miyagi 980-8578 Japan; 3 Department of Pathology and Histotechnology, Tohoku University Graduate School of Medicine, 2-1 Seiryo-machi, Aoba-ku, Sendai, Miyagi 980-8578 Japan; 4 Department of Surgical Oncology, Tohoku University Graduate School of Medicine, 2-1 Seiryo-machi, Aoba-ku, Sendai, Miyagi 980-8578 Japan

**Keywords:** Breast cancer, Microenvironment, Cancer-associated adipocytes, S100A7

## Abstract

**Background:**

Breast adipocytes play important roles in both the development and function of mammary epithelial cells. Therefore, carcinoma–adipose stromal cell (ASC) interactions have been considered pivotal in supporting tumor growth in breast cancer. In addition, it has been demonstrated that the biological features of cancer-associated adipocytes differ from those of normal ASCs. Therefore, we investigated an interaction between ASCs and carcinoma cell lines to identify genes associated with ASC invasion of carcinoma cells.

**Methods:**

3T3-L1 ASC-derived conditioned medium (CM) was treated to measure the proliferation rate of breast cancer cells. To further examine the effect of ASCs, breast cancer cells were cocultivated with either primary human or 3T3-L1 ASCs for migration assays, DNA microarrays, quantitative real-time polymerase chain reactions, and Western blotting experiments. Furthermore, immunoreactivity of S100A7, the most upregulated gene in MCF7, after coculture with ASCs was evaluated for 150 breast cancer tissues to statistically analyze its association with clinicopathological parameters.

**Results:**

We first confirmed that ASC-derived CM treatment enhanced the cell proliferation rate of MCF7, T47D, SK-BR-3, and ZR-75-1 cell lines, whereas the migration rate of breast cancer cells was promoted by coculture with ASCs. We identified that a small calcium-binding protein, S100A7, was markedly upregulated (by 5.8-fold) in MCF7 cells after coculture with primary human ASCs. Knockdown of S100A7 significantly suppressed ASC-stimulated cell proliferation and migration rate, indicating a possible involvement of S100A7 in the carcinoma–ASC interaction in breast tumors. Furthermore, strong S100A7 immunoreactivity was detected at the invasive front of adipose stromal tissues compared with that at the intratumoral area. The status of S100A7 was also significantly correlated with adverse pathological parameters, and multivariate analysis revealed that S100A7 could be an independent prognostic marker for a poor relapse-free survival rate. Moreover, induction of oncostatin M was detected in cancer-stimulated ASCs, whereas the downstream S100A7 binding proteins/receptor for advanced glycation endproducts were significantly upregulated in correspondence with S100A7 expression in breast cancer cells after coculture with ASCs.

**Conclusions:**

The results of our study suggest that paracrine production of cytokines from ASCs stimulates breast carcinoma cell growth via upregulation of S100A7 expression in breast cancer cell lines.

**Electronic supplementary material:**

The online version of this article (doi:10.1186/s13058-017-0863-0) contains supplementary material, which is available to authorized users.

## Background

In breast cancer, the proliferation and invasion of carcinoma cells are not only determined by epigenetic changes of these cells but also greatly influenced by the cells’ own tissue microenvironment and their interaction with stromal cells. For instance, cancer-associated fibroblasts (CAFs) and/or tumor-associated macrophages have previously been demonstrated to promote carcinoma cell survival and invasiveness by secreting cytokines, growth factors, chemokines, and proteases [1, 2]. Although the most abundantly present stromal component in the breast is well known to be adipose stromal cells (ASCs), the significant roles of breast ASCs in the development of mammary epithelial cells have been demonstrated in a number of studies on mammary fat pads in experimental animal models [3, 4]. In addition, mammary fat pads are a source of not only lipids but also hormones, growth factors, extracellular matrix, and adipocytokines [4, 5]. However, the potential interactions between carcinoma cells and ASCs are largely unexplored.

A number of epidemiological studies have demonstrated a link between fat distribution, overweight, or obesity and breast cancer risk, though the results could vary depending on menopausal status, ethnicity, and anthropometry [6–10]. ASCs have been reported as sites of estrogen production via aromatase, and the link between risks of breast cancer and obesity/adiposity among postmenopausal women with the luminal type have been reasonably explained [11–13]. However, little is known about the mechanism of ASC-stimulated tumorigenesis independent of estrogenic action. Coinjection of adipose-derived stem cells and MDA-MB-231 cells into mouse mammary fat pads resulted in more frequent local tumor invasion than that occurring in MDA-MB-231 cells alone [14]. Histological observations have indicated that ASCs adjacent to invasive carcinoma cells tend to be smaller in size than those farther from the carcinoma [15]. Furthermore, murine ASCs have been shown to exhibit fewer lipid droplets, decreased late-response adipose marker expression, and overexpression of inflammatory cytokines and proteases following interaction with breast carcinoma cells [15–17]. On the basis of these phenotypic alterations, these ASCs have been referred to as *cancer-associated adipocytes* (CAAs) [18]. However, a global change of gene expressions has not been explored linking the interaction between invasive breast carcinoma cells and CAAs.

Given that ASCs undergo phenotypic changes upon interacting with carcinoma cells, this raises the question of how carcinoma cells might be influenced by ASCs. In this study, we hypothesized that ASC-derived factors could promote the growth and migration of breast carcinoma cells by alteration of gene expression. Therefore, we first examined a possible influence of ASC-derived factors on breast carcinoma cells with an experiment involving conditioned medium (CM) treatment and coculture. Microarray analysis demonstrated that S100A7 was significantly upregulated in MCF7 cells following coculture with primary human ASCs. S100A7 is known to be overexpressed in psoriasis as well as in squamous cell carcinomas such as those of the lung and breast [19]. We therefore further examined the roles of S100A7 using immunohistochemistry to analyze its association with clinicopathological parameters, in addition to its potential association with S100A7 inducers and downstream oncogenic pathways involved in carcinoma–ASC interactions.

## Methods

### Cell culture

Human breast carcinoma cell lines MCF7, T47D, ZR-75-1, and SK-BR-3 and murine 3T3-L1 preadipocytes (pre-3T3) were obtained from the American Type Culture Collection (Manassas, VA, USA). All these cell lines were maintained in high-glucose DMEM supplemented with 10% FBS and 1% penicillin-streptomycin. Cell lines purchased 6 months before the study were tested for cross-contamination by using the short tandem repeat profiling method, and in all the in vitro experiments they were used within ten passages. Differentiation of pre-3T3 was induced at 2 days after reaching confluence by replacing the media supplemented with 3-isobutyl-1-methylxanthine (IBMX), dexamethasone (DEX), and insulin in accordance with the manufacturer’s protocol (Cayman Chemical Company, Ann Arbor, MI, USA). After the induction, DMEM supplemented with insulin was replaced every 2–3 days until lipid droplets were grown in more than 80% of the cells.

Primary human subcutaneous preadipocytes (human primary adipose stromal cells [pre-hASCs]) PT-5020 (female, 38 years old, body mass index [BMI] 26 kg/m^2^) were purchased from Lonza (Basel, Switzerland). The pre-hASCs were grown to confluence in preadipocyte basal medium 2 (PBM-2; Lonza) and differentiated by replacement with differentiation medium (insulin, DEX, IBMX, and indomethacin in PBM-2) as described in the manufacturer’s protocol. Differentiated 3T3-L1 adipocyte stromal cells (3T3a) and human adipocyte stromal cells (hASCs) were stained with Oil Red O stain to quantify the lipid ratio, as shown in Additional file 1 and Additional file 2: Figure S1. In our study, 3T3a cells and hASCs were used as a model of ASCs.

Either murine or human preadipocytes/ASCs were cocultured with breast cancer cells using a 2D coculture method with DMEM, as shown in Additional file 2: Figure S1C and as described in a previous report [20]. The two types of cells were separately cultured in different compartments interposed by a 0.4-μm-pore membrane (ThinCerts^TM^; Greiner Bio-One, Frickenhausen, Germany), through which only soluble factors can travel.

### Conditioned medium treatment and proliferation assay

To make CM from each cell line (breast cancer cells, preadipocytes, and ASCs), FBS-free and phenol-free DMEM was replaced for 24 h after being washed twice with PBS. Each medium (breast cancer cell-derived CM, preadipocyte-derived CM, and adipose stromal cell -derived conditioned medium [ACM]) was then collected and filtered to remove the dead or floating cells with 0.25-μm-pore filter before it was concentrated by centrifugation using a 3 kDa filter centrifuge at 6000 rpm for 90 minutes. Each cell line was treated with CM (100 μg/ml) for 4 days. For repetitive purposes, 3T3-L1 was used instead of finite primary human ASCs in this experiment as an ASC model. The number of cells was counted using a WST-8 colorimetric assay (Cell Counting Kit-8; Dojindo Laboratories, Kumamoto, Japan).

### Migration assay

Breast cancer cells were seeded onto an 8.0-μm-pore transparent polyethylene terephthalate membrane (Corning Inc., Corning, NY, USA) with serum-free DMEM and inserted into 24-well plates with or without 3T3-L1 ASCs in 10% FBS DMEM. After 24 h of coculture, cells on the inner membrane of the insert were removed and fixed with 100% methanol for 5 minutes, followed by hematoxylin staining for 25 minutes. Each membrane with nuclear staining was divided into four sections, and cells were counted under a light microscope at × 200 magnification for each field. The total numbers of cells were averaged (*n* = 3) for comparison with other conditions.

### Quantitative real-time polymerase chain reaction

Total RNA was extracted using TRIzol reagent, and complementary DNA was synthesized using a QuantiTect Reverse Transcription Kit (QIAGEN, Hilden, Germany). Quantitative real-time polymerase chain reaction (qRT-PCR) was carried out using the LightCycler System (Roche Molecular Diagnostics, Pleasanton, CA, USA) and the QuantiFast SYBR Green PCR Kit (QIAGEN). The expression was normalized with β-actin messenger RNA (mRNA) expression levels. The sequences of primers used for qRT-PCR are listed in Additional file 1: Table S1. PCR products were electrophoresed, stained with ethidium bromide, and imaged using the ChemiDoc^TM^ XRS+ System (Bio-Rad Laboratories, Hercules, CA, USA). Band intensities were measured using Image Lab^TM^ Image capture and analysis software (Bio-Rad Laboratories). Three independent assays were performed with triplicate samples.

### Microarray analysis

A Low Input Quick Amp Labeling Kit (Agilent Technologies, Santa Clara, CA, USA) was used for the preparation of cyanine-3-labeled RNA (cRNA) from MCF7 cells cocultivated with either pre-hASCs or hASCs for comparison with monocultured MCF7 cells. cRNAs were applied to the slides (SurePrint G3 Human GE 8 × 60 K; Agilent Technologies) and hybridized for 17 h as instructed in the manufacturer’s protocol. Scanned data were interpreted using GeneSpring version 12.2 software (Agilent Technologies) by clustering and gene ontology analysis.

### Immunoblotting

Cells were dissolved in M-PER™ Mammalian Protein Extraction Reagent Nonidet P-40 buffer with Halt Protease Inhibitor Cocktail and Phosphatase Inhibitor (Pierce Biotechnology, Rockford, IL, USA), and the protein concentration was determined using a protein assay kit (Wako Pure Chemical Industries, Osaka, Japan). Proteins were resolved by SDS-PAGE and transferred onto a polyvinylidene difluoride membrane. After blocking with 5% nonfat dry skim milk for 1 h at room temperature, membranes were incubated overnight at 4 °C with primary mouse monoclonal antibodies for anti-S100A7 (H-8, sc-377084; Santa Cruz Biotechnology Inc., Dallas, TX, USA) at 1:100 dilution, anti-receptor for advanced glycation endproducts (anti-RAGE) (ab54741; Abcam, Cambridge, UK) at 1:1000 dilution, and anti-oncostatin M (anti-OSM) (17001; R&D Systems, Minneapolis, MN, USA) at 1:200 dilution. Following rinses using Tris-buffered saline with Tween 20, the membranes were incubated for 1 h with HRP-conjugated antimouse immunoglobulin G (GE Healthcare, Little Chalfont, UK) at room temperature. The protein bands were visualized by using enhanced chemiluminescence (ECL Prime Western Blotting Detection Reagents; GE Healthcare) and the ChemiDocTM XRS+ System.

### Small interfering RNA transfection

Small interfering RNAs (siRNAs) for S100A7 with two different sequences (1 and 2) were purchased from Sigma-Aldrich (St. Louis, MO, USA). The target sequences against *S100A7* siRNAs are described in Additional file 1: Table S2. Each cancer cell line was treated with 5nM *S100A7* siRNA 48 h prior to the assay and replaced when the CM treatment for the proliferation assay and migration assay was conducted.

### Breast cancer tissue specimens

A total of 150 invasive ductal breast carcinoma tissues were retrieved from Japanese females (mean age 55.6 ± 12.9 years, range 27–87) who had undergone surgery between 2004 and 2008 at Tohoku University Hospital (Sendai, Japan). All the specimens were fixed in 10% formalin and embedded in paraffin wax.

### Immunohistochemistry

Immunohistochemistry was performed by the streptavidin-biotin amplification method using a Histofine kit (Nichirei Biosciences, Tokyo, Japan) as described previously [21]. Mouse monoclonal antibody for Ki-67 (MIB1) was purchased from Dako (Carpinteria, CA, USA), and S100A7 (47C1068) was purchased from LifeSpan BioSciences, Inc. (Seattle, WA, USA). The primary antibodies of Ki-67 (1:300) and S100A7 (1:3000) were applied following antigen retrieval by autoclaving the slides at 120 °C for 5 minutes in citric acid buffer (pH 6.0). Immunoreactivity was then visualized with the 3,3-diaminobenzidine colorimetric reaction, and all the sections were counterstained with hematoxylin. For the estrogen receptor (ER) and progesterone receptor (PR), anti-ER (SP1) and anti-PR (1E2) were purchased from Roche Diagnostics Japan (Tokyo, Japan) for automated immunohistochemistry using the Ventana Benchmark XT platform (Roche Diagnostics Japan). Detection of human epidermal growth factor receptor 2 (HER2) immunoreactivity was done using the HercepTest assay (Dako).

The evaluation and scoring of Ki-67, ER, PR, and HER2 immunoreactivity was performed as described in a previous report [21]. The cytoplasmic/nucleus immunoreactivity of S100A7 was categorized into two groups, negative and positive, on the basis of relative immunointensity and area of S100A7 (positive = more than 1% detected, negative = less than 1% detected) as described in a previous report [22]. The results of immunohistochemical analysis were independently evaluated by two of the authors (MS and YM), and the cases for which there was disagreement between the observers were reevaluated by the observers together using multiheaded light microscopy.

### Statistical analysis

The results were expressed as the mean ± SD. Statistical analysis was performed using JMP Pro 11.0.0 software (SAS Institute, Cary, NC, USA). The statistical difference of the two groups was determined by Student’s *t* test, and multiple groups were analyzed by one-way analysis of variance. Fisher’s exact test was used for the contingency table. A survival curve was generated according to the Kaplan-Meier method, and statistical significance was calculated using the log-rank test. *P <* 0.05 was considered statistically significant.

## Results

### Characteristics of ASCs were changed following interaction with breast carcinoma cells

**Fig. 1:**
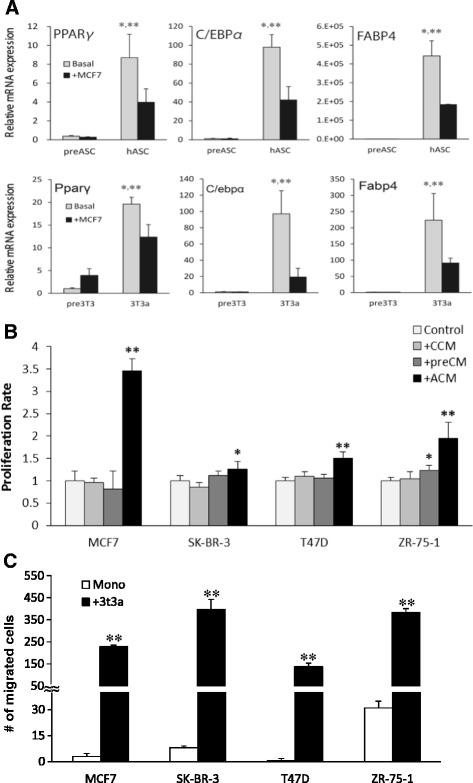
Carcinoma–adipose stromal cell (ASC) interaction alters characteristics of ASCs and promotes proliferation and migration rate of breast cancer cells. **a** Reduction of adipocyte transcription markers in human primary adipocyte stromal cells (hASCs) and 3T3-L1 adipocyte stromal cells (3T3a) by coculture with MCF7 cells, from those levels in preadipocytes. *Pre-hASCs* Primary human preadipocytes. * *P* < 0.0001 compared with basal preadipocytes; ** *P* < 0.05 compared with hASC + MCF7. **b** Cell proliferation rate by WST-8 assay in MCF7, T47D, SK-BR-3, and ZR-75-1 cells by treatment of conditioned medium (CM) derived from each cell line, pre-3T3-L1, and 3T3a for 4 days. *CCM* Breast cancer cell-derived conditioned medium, *pre-CM* Preadipocyte-derived conditioned medium, *ACM* 3T3a adipose stromal cell-derived conditioned medium. * *P <* 0.05; ** *P <* 0.0001 compared with control. **c** Coculture with ASCs (+3T3a) significantly induced migration rate of MCF7, SK-BR-3, T47D, and ZR-75-1 cells compared with monocultured cells. * *P <* 0.05 compared with monoculture. *C/EBPα* CCAAT/enhancer-binding protein α, *FABP4* Fatty acid-binding protein 4, *mRNA* Messenger RNA, *PPARγ* Peroxisome proliferator-activated receptor-γ

We first examined the expression of adipogenic transcription markers in both human primary and murine ASCs to explore how characteristics of ASCs could be modified in our coculture model. Of note, we selected 3T3-L1 fibroblasts as a stable and replicable adipocyte-differentiating cell line on the basis of a number of previous studies on coculture with human breast cancer cell lines [17, 23, 24]. Previously described adipogenic markers were examined as adipocyte markers, including peroxisome proliferator-activated receptor-γ (PPARγ), which regulates adipocyte differentiation; CCAAT/enhancer-binding protein α (C/EBPα), responsible for mitotic growth arrest and late-stage differentiation; and fatty acid-binding protein 4 (FABP4), known for fatty acid intake, transport, and metabolism [25, 26]. The expression of these adipocyte markers was markedly increased in both murine and human ASCs, as expected, but it was significantly reduced by cocultivation with MCF7 (Fig. 1a). In addition, cancer-derived CM treatment significantly reduced the ratio of ASC lipid droplets (Additional file 2: Figure S1D). These phenotypic changes provided additional evidence for previously described characteristics of CAAs following stimulation by carcinoma cells.

### ASC-derived factors induced carcinoma cell proliferation and migration rate

Alteration of ASCs following the interaction with carcinoma cells indicated the possible modification of the biological behavior in carcinoma cells. Thus, we examined the effects of adipocyte-derived factors on breast cancer cell proliferation and migration. After the treatment of ACM at 100 μg/ml for 96 h, the proliferation rates of the MCF7, SK-BR-3, T47D, and ZR-75-1 cells were significantly increased compared with CM from cancer cells and preadipocytes, suggesting that mature adipocytes rather than preadipocytes are in fact contributing with cancer cell progression (Fig. 1b). Similarly, coculture with ASCs significantly promoted the migration rate in MCF7 and SK-BR-3, T47D, and ZR-75-1 cells (Fig. 1c).

### Interaction with CAAs upregulated S100A7 expression in breast cancer cells

**Fig. 2:**
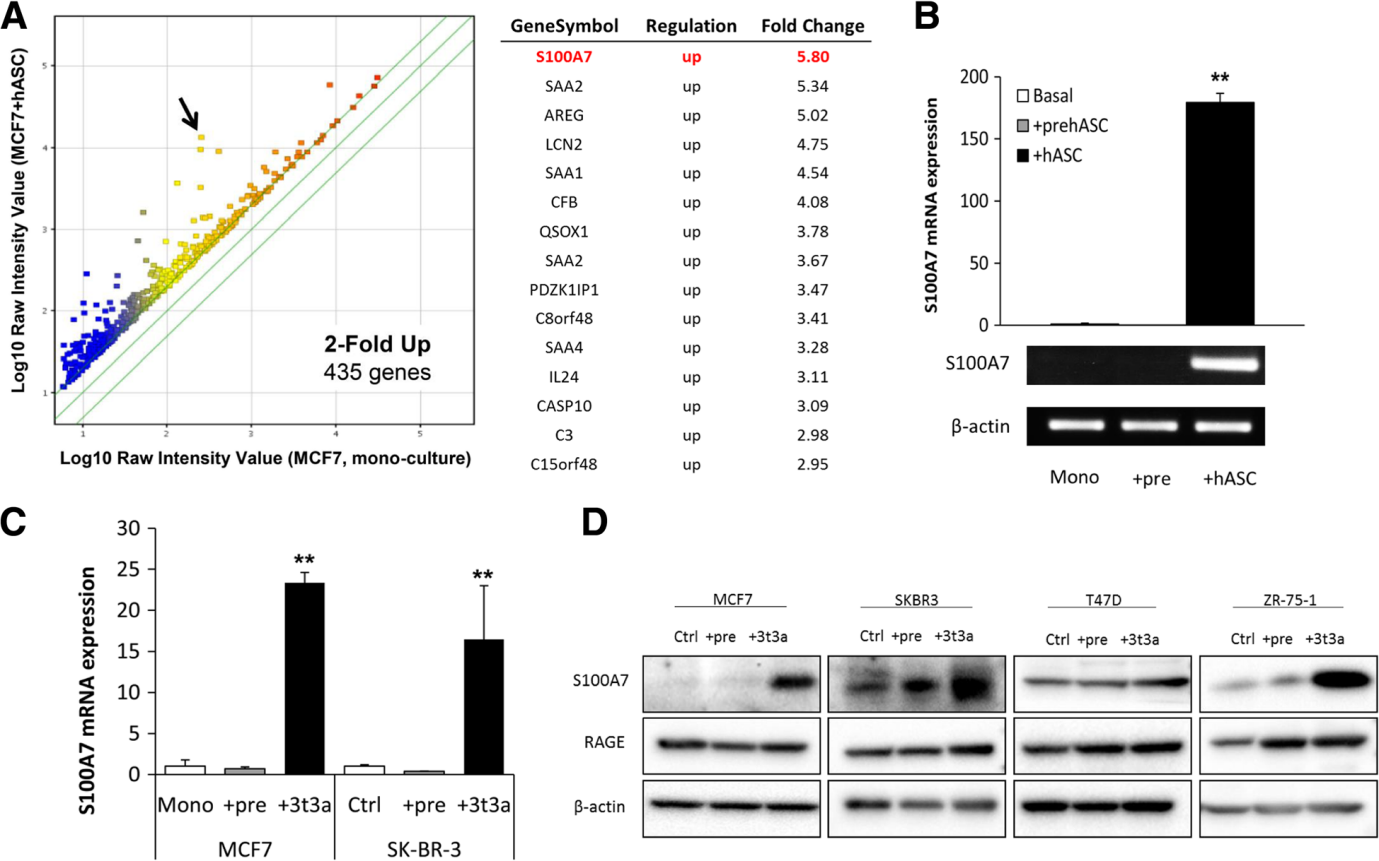
S100A7 expression was significantly upregulated in breast cancer cells by interaction with adipose stromal cell (ASCs). **a** Scatterplot analysis of microarray data indicates a total of 435 genes with more than a twofold increase in MCF7 + human adipocyte stromal cells (hASCs) compared with MCF7 cell monoculture are shown above the *diagonal line*. The table at *right* shows the top 15 upregulated genes among the group of genes in the scatterplot. **b** Verification of S100A7 messenger RNA (mRNA) expression by quantitative real-time polymerase chain reaction in MCF7 cells cocultivated with primary human preadipocytes/ASCs compared with monoculture. **c** Verification of S100A7 mRNA in MCF7 and SK-BR-3 cells cocultured with 3T3-L1 preadipocytes/ASCs compared with monoculture. **d** Protein expression levels of S100A7 and receptor for advanced glycation endproducts in MCF7, SK-BR-3, T47D, and ZR-75-1 cells cocultivated with 3T3-L1 preadipocytes/ASCs compared with monoculture. ** *P* < 0.0001 compared with control. The data shown are representative of three independent experiments and shown as mean ± SD

The induction of carcinoma cell proliferation and migration by ASC-derived factors suggested the possibility of DNA expression pattern changes in carcinoma cells after ASC stimulation. Microarray analysis revealed that 435 genes were upregulated (greater than twofold) in MCF7 + hASCs compared with monocultured MCF7 cells (Fig. 2a). Overexpression of the most upregulated gene (at 5.8-fold), a small calcium-binding protein (S100A7), was subsequently confirmed by qRT-PCR (Fig. 2b). We also confirmed elevated S100A7 mRNA expression in primary breast tumor cells compared with the normal epithelial cells, both retrieved from frozen primary human breast cancer tissues with tumor infiltration into surrounding adipose tissues (Additional file 3: Figure S2). For the purpose of the following experiments, S100A7 upregulation by coculture with murine ASCs was also confirmed in different types of breast cancer cell lines relative to that in the pre-3T3 or monoculture at mRNA and protein levels (Fig. 2c and d). The results of the 2D invasion assay and S100A7 immunostaining also indicated that ASCs adjacent to the carcinoma cells could promote S100A7 expression. Higher expression of S100A7 was detected at the invasive front (IF) of the MCF7 cells after the removal of the silicon chamber that separated 3T3a and MCF7 (Additional file 4: Figure S3). Notably, marked immunoreactivity of S100A7 was detected in morphologically altered MCF7, and lipid droplets embedded in the MCF7 cells were discernible after exposure to ASCs (Additional file 4: Figure S3).

### S100A7 regulatory signaling molecules were also induced by CAA–cancer cell interaction

OSM has previously been reported to induce S100A7 expression in breast cancer cells [27]. In our experimental model, a significant increase of OSM expression was detected in ASCs followed by coculture with MCF7, T47D, and ZR-75-1 cells compared with monocultured ASCs (Additional file 5: Figure S4). Elevated expression levels of S100A7 then promote breast cancer malignancy via several signaling pathways, such as RAGE [28, 29]. Our results show that expression of RAGE was increased in breast cancer cells in correspondence with the elevated S100A7 expression by coculture with ASCs (Fig. 2d).

**Fig. 3:**
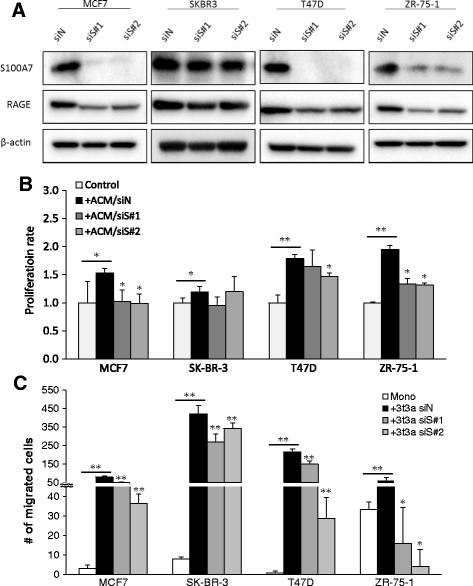
S100A7 expression knockdown significantly decreased adipose stromal cell (ASC)-derived factors induced proliferation and migration rate in breast carcinoma cells. **a** S100A7 and receptor for advanced glycation endproducts (RAGE) protein expression levels were significantly reduced by 5 nM small interfering RNA (siRNA) treatment after 72 h. Knockdown of S100A7 expression suppressed adipose stromal cell-derived conditioned medium (ACM) or adipocyte coculture induced (**b**) proliferation and (**c**) migration rate in MCF7, SK-BR-3, T47D, and ZR-75-1 cells. *siN* Negative control small interfering RNA, *siS* S100A7 small interfering RNA, *ctrl* Control, *Mono* Monoculture, *+3t3a* Coculture with 3T3-L1 adipose stromal cells. * *P <* 0.05 compared with siN + ACM/3T3a; ** *P <* 0.0001 compared with control

To further explore a role of S100A7 expression in carcinoma–ASC interaction, we examined the proliferation and migration rates of MCF7, SK-BR-3, T47D, and ZR-75-1 cells stimulated by ASC-derived factors by suppressing S100A7 expression. Successful knockdown of *S100A7* siRNAs 1 and 2 significantly reduced expression of RAGE, as well as ACM-induced proliferation and migration rates in MCF7, SK-BR-3, T47D, and ZR-75-1 cells (Fig. 3a–c). The slight difference in the inhibitory effect of S100A7 siRNA indicated that cellular phenotypes of carcinoma cells could be partially affected by the nonspecific toxicity with a combination of cell culture conditions as previously reported [30]. These results suggest that the regulation of S100A7 expression mediates ASC-stimulated breast cancer progression.

### S100A7 immunoreactivity was enhanced at invasive front of breast adipose stromal tissues and was associated with poor prognosis in patients with breast cancer

**Fig. 4:**
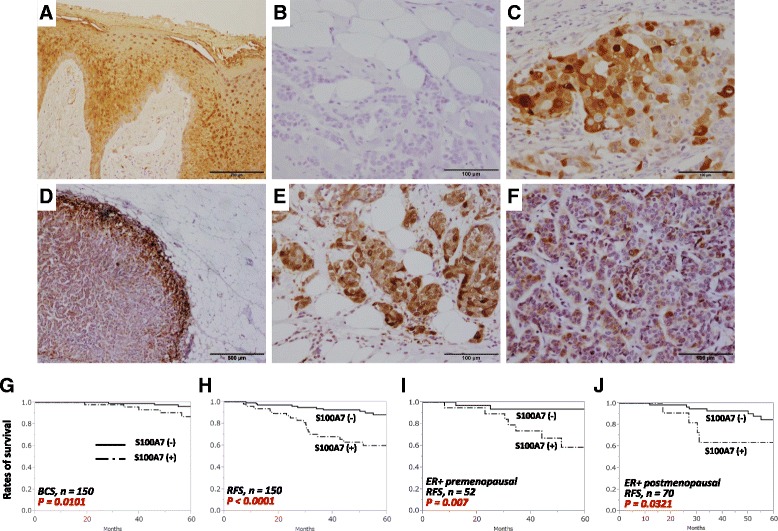
S100A7 immunoreactivity and prognosis in human breast carcinoma tissues. S100A7 immunoreactivity in (**a**) inflamed epidermis of skin tissue as a positive control, showing (**b**) positive (*n* = 47) and (**c**) negative cases (*n* = 103) in human breast cancer tissues. **d** Different expression patterns of S100A7 were detected between the invasive front of surrounding adipose tissues and the intratumoral area. Strong immunoreactivity was detected in carcinoma cells (**e**) at the invasive front compared with (**f**) the intratumoral area with weak immunoreactivity. Kaplan-Meier curve according to S100A7 status indicating (**g**) breast cancer-specific survival (BCS) rate and (**h**) relapse-free survival (RFS) rate for all the patients, as well as RFS rate of (**i**) premenopausal (S100A7^+^, *n* = 20; S100A7^−^, *n* = 32) and (**j**) postmenopausal (S100A7^+^, *n* = 11; S100A7^−^, *n* = 59) patients with estrogen receptor-positive (ER^+^) status. *P* values were determined by log-rank test

We then investigated clinical or biological roles of S100A7 expression among 150 Japanese patients with invasive ductal breast carcinoma by immunohistochemistry. S100A7 immunoreactivity was detected in the cytoplasm and/or nucleus of 47 cases (31.3%) (Fig. 4a–c). Among the S100A7^+^ group, immunoreactivity was detected both in the intratumoral (IT) region and at the adipose stromal tissue IF for 31 cases (66.0%), with 6 cases (12.8%) only in the IT region and 5 cases (10.6%) only in IF; 5 cases (10.6%) had no adipose tissue invasion. Notably, seven cases exhibited more pronounced S100A7 immunoreactivity at IF than in the IT region among the IT^+^/IF^+^ group (Fig. 4d–f).

Statistical analysis revealed that S100A7 status was positively correlated with stage (*P* = 0.016), Ki-67 labeling index (LI) (*P* < 0.0001), histological grade (*P* = 0.027), and lymph node metastasis (*P* = 0.038) in the patients examined, and that it was negatively associated with ER (*P* = 0.001) and PR (*P* = 0.001) status (Table 1). Kaplan-Meier analysis also demonstrated that the S100A7^+^ group was significantly associated with poor clinical outcome in terms of both breast cancer-specific survival (*P* = 0.0101) and relapse-free survival (RFS) (*P* < 0.0001) (Fig. 4g and h). Multivariate analysis revealed that S100A7 could be an independent prognostic marker for RFS (Table 2). Further analysis of RFS with ER^+^ premenopausal and postmenopausal patients showed similar results (*P* = 0.0007 and *P* = 0.0321, respectively) (Fig. 4i and j). The presence of S100A7 immunoreactivity was also significantly associated with RFS (*n* = 3455, *P* = 0.0085) and overall survival rate (*n* = 1115, *P* = 0.053) in a larger cohort derived from a publicly available online database (Additional file 6: Figure S5a). However, no significant association was detected between S100A7 and RFS (*n* = 788, *P* = 0.78) or for overall survival rate (*n* = 292, *P* = 0.38) when the objective was limited to ER^−^ cases (Additional file 6: Figure S5b).

**Table 1:** Statistical analysis of S100A7 immunoreactivity and clinicopathological parameters

Pathological parameters	S100A7 immunoreactivity		*P* value
	Negative (−)	Positive (+)	
	*n* = 103	*n* = 47	
Age, years	56.6 ± 1.3	53.4 ± 1.9	0.149
Menopausal status			
Premenopausal	37 (24.7%)	22 (14.7%)	0.213
Postmenopausal	66 (44.0%)	25 (16.7%)	
Stage			
I	58 (39.2%)	18 (12.2%)	**0.016**
II	35 (23.6%)	16 (10.8%)	
III	9 (6.1%)	12 (8.1%)	
Estrogen receptor status			
Positive	90 (60.8%)	31 (20.9%)	**0.0012**
Negative	11 (7.4%)	16 (10.8%)	
Progesterone receptor status			
Positive	64 (43.2%)	16 (10.8%)	**0.0013**
Negative	37 (25.0%)	31 (20.9%)	
HER2*/neu* status			
Positive	9 (6.1%)	7 (4.7%)	0.272
Negative	92 (62.2%)	40 (27.0%)	
Ki-67 LI	12.1 ± 1.2	21.3 ± 1.7	**<0.0001**
Histological grade			
1, 2 (well, moderate)	79 (54.1%)	21 (14.4%)	**0.027**
3 (poor)	28 (19.2%)	18 (12.3%)	
Lymph node metastasis			
Positive	27 (18.2%)	21 (14.2%)	**0.038**
Negative	74 (50.0%)	26 (17.6%)	

*HER2* Human epidermal growth factor receptor 2, *LI* Labeling index

*P* values (*P <* 0.05) were determined by analysis of variance and Fisher’s exact test

Significant *P* values (*P* < 0.05) were indicated in bold

**Table 2:** Univariate and multivariate analysis of S100A7 expression

Clinical parameters	Univariate		Multivariate	
	*P* value	Relative risk (95% CI)	*P* value	Relative risk (95% CI)
Relapse-free survival				
Ki-67 LI (≥15%/<15%)	**<0.0001**	**7.3 (3.0–21.7)**	**0.0004**	**5.7 (2.1–18.1)**
S100A7 status (+/−)	**0.0005**	**3.8 (1.8–8.4)**	**0.0227**	**2.5 (1.1– 5.9)**
Lymph node metastasis (+/−)	**0.0006**	**3.6 (1.7–8.0)**	**0.0150**	**2.8 (1.2–6.6)**
Histological Grade (3/1, 2)	**0.0357**	**2.3 (1.1–4.8)**		
HER2 status (+/−)	0.0861			
ER status (−/+)	0.0998			
Menopausal Status (pre/post)	0.4209			
Breast cancer survival				
Ki-67 LI (≥15%/<15%)	**0.0009**	**13.0 (2.5–239.2)**	**0.0346**	**7.5 (1.1–150.8)**
Lymph node metastasis (+/−)	**0.0098**	**5.1 (1.5–23.1)**		
S100A7 status (+/−)	**0.0124**	**4.7 (1.4–18.1)**		
Histological Grade (3/1,2)	**0.0127**	**4.6 (1.4–17.6)**		
ER status (+/−)	0.1371			
Menopausal Status (pre/post)	0.3919			
HER2 status (+/−)	0.6477			

Data considered significant (*P <* 0.05) are shown in boldface type. Parameters with significant values were further examined in the multivariate analysis. Relative risks of only significant values are shown

*Abbreviations: ER*, Estrogen receptor, *HER2* Human epidermal growth factor receptor 2, *LI* Labeling index

## Discussion

In recent decades, obesity and a higher BMI (>25 kg/m^2^) have become established as major health risk factors for cardiovascular disease; type 2 diabetes; and various cancers, including breast cancer [31–34]. Recent preclinical studies have indicated that obesity and a high BMI were significantly associated with poor prognosis of patients with breast cancer [32]. The clinical and biological importance of breast adipose tissue has been well documented in ER^+^ breast cancer as a site of estrogen biosynthesis through the activation of aromatase [35]. However, its roles in the breast cancer microenvironment remain virtually unexplored.

In the present study, we have demonstrated that interaction between ASCs and breast carcinoma cells significantly changed the biological behavior of both ASCs and carcinoma cells. In agreement with previous mouse studies, PPARγ, C/EBPα, and FABP4 expression levels were markedly increased after differentiation of pre-hASC/pre3T3 cells, whereas these adipogenic markers were significantly reduced in hASC/3T3a cells following coculture with MCF7 cells [17, 26, 36, 37]. Elevated expression levels of multiple inflammatory cytokines were also detected in ASCs cocultured with carcinoma cells (data not shown). These phenotypic changes provide additional evidence for previously reported CAA characteristics or the modification of ASCs following an interaction with carcinoma cells. We also observed a significant increase in the breast carcinoma cell proliferation rate upon ACM treatment and in the migration rate upon coculture with ASCs, indicating that signaling molecules produced from ASCs in the tumor microenvironment promote tumor progression. Despite the fact that CAAs resemble a fibroblast-like phenotype, the exact origins of the cells remain undetermined [38, 39]. In contrast, CAFs could be recruited from nonmalignant stromal cells by cancer cells in breast cancer tissues [38, 39]. Further investigations are necessary to clarify whether the CAAs could be formed as a result of dedifferentiation or delipidation from mature cells or as newly recruited cells of mesenchymal origin.

Comprehensive microarray analysis in our study revealed that *S100A7* (psoriasin) could be a potent regulatory gene involved in the carcinoma–ASC interaction. Enhanced expression of S100A7 has been recognized in psoriatic skin lesions, as well as in squamous cell tumors of the skin, mouth, lung, and breast [40–43]. In the present study, S100A7 was overexpressed in breast carcinoma cells following interaction with hASC/3T3a cells but not with pre-hASC/pre-3T3 cells. We also demonstrated that *S100A7* knockdown inhibited ASC-derived factor-induced cell proliferation and migration of various breast cancer cells. The variance of inhibitory effects by *S100A7* knockdown among breast cancer cells indicated that S100A7 may interact with several downstream proteins, such as c-Jun activation domain-binding protein 1 or Ran-binding protein M, to regulate carcinoma cell proliferation [44–46]. Other mechanisms associated with the genes detected, as shown in Fig. 1a, could also be involved in the process, in addition to S100A7 signaling pathways. For instance, the fourth most upregulated gene (Fig. 1a), lipocalin 2, has also been reported to be oncogenic in both lung and breast cancers and could be stimulated by interferon-γ and tumor necrosis factor-α in murine subcutaneous adipocytes [47–49]. Further investigations are required to clarify what other factors or mechanisms are involved in cancer–ASC interaction. In this study, however, the clinicopathological significance of S100A7 in human breast cancer tissues supported the hypothesis that ASC-stimulated S100A7 could play important roles in tumorigenesis in a subset of patients with breast cancer. Of particular interest, lipid droplets were detected in S100A7-expressing MCF7 cells following exposure to ASCs. It has been reported that S100A7 forms a complex with FABP4 in human keratinocytes, and adipocyte-derived lipids have previously been shown to promote tumor growth by providing an energy source such as FABP4 in ovarian cancer [50, 51]. Taken together, S100A7 expression at the site of breast carcinoma invasion into adipose stromal tissue could represent the status of carcinoma–ASC interaction in a breast tumor microenvironment.

Accumulated evidence shows that S100A7 overexpression is associated with breast cancer malignancy. However, to the best of our knowledge, this is the first study to present its intratumoral distribution among patients with invasive breast carcinoma. The more pronounced S100A7 immunoreactivity of breast carcinoma cells at the IF of adjacent adipose stromal tissues compared with that in the IT region suggests that upregulation of S100A7 could be promoted when carcinoma cells interact with breast ASCs. In concordance with the results of our in vitro study, S100A7 was positively correlated with malignant markers such as higher Ki-67 LI, advanced clinical stage, higher histological grade, and the presence of lymph node metastasis. The association of S100A7 status with ER^−^/PR^−^ status is also consistent with the results of a Canadian cohort study [22]. Kaplan-Meier and multivariate analyses of Japanese patients (*n* = 150) and a public database (*n* = 3455) also suggested that S100A7 could be an independent marker of poor prognosis for the recurrence of invasive breast carcinoma, but the correlation did not remain significant when the patients were limited to the ER^−^ group. This may be due in part to S100A7 activation being promoted when the main driving force of tumorigenesis, estrogen signaling, is silenced by estrogen depletion or drug treatment. It is also suggested that hormone status may not be directly linked to S100A7 prognosis, because its RFS retains the same tendency in both the ER^+^ pre- and postmenopausal groups. However, further investigation is required to elucidate the relationship between S100A7 and ER status with regard to cancer–ASC interaction.

S100A7 expression has been reported to be consistently induced in MCF7, T47D, and MDA-MB-468 cells by exogenous stimulation of cytokines such as OSM and interleukin (IL)-6 [27]. Lapeire and colleagues demonstrated that OSM-neutralizing antibodies in combination with adipose tissue-derived CM treatment abolished the breast cancer malignant signatures and suggested that OSM is a more relevant factor in stimulation of breast cancer progression than other cytokines, such as IL-6 [52]. They also provided data suggesting that activation of several signaling cascades, such as signal transducer and activator of transcription 3 (STAT3), phosphoinositide 3-kinase, and extracellular signal-regulated kinase 1/2, were involved in OSM induction of S100A7 upregulation. In agreement with previous findings in terms of a linkage between OSM/STAT signaling in CAA and S100A7 induction by CAA-derived CM in MCF7, our results further confirm the importance of paracrine production of OSM in cancer-stimulated ASCs, which could promote breast cancer progression via S100A7 induction.

There are several possible mechanisms for S100A7-mediated downstream regulation of tumorigenesis, and RAGE has previously been shown to bind directly with S100A7 for its activation [53, 54]. Though increased expression levels of S100A7 and RAGE have been frequently observed in ER^−^ breast cancer, our results demonstrate that S100A7 and RAGE expression levels could also be induced in ER^+^ breast cancer cells by an interaction with ASCs. As previous reports and our results demonstrate, expression of RAGE is modulated by S100A7 expression status. Thus, it is suggested that RAGE might be activated in CAA-stimulated breast cancer cells via upregulation of S100A7 expression.

## Conclusions

We have shown that ASC-derived factors significantly promoted carcinoma cell proliferation and migration in breast carcinomas, and we have identified a potential oncogene, *S100A7*, that was markedly upregulated by an interaction between carcinoma cells and CAAs. Our findings also suggest that S100A7 could be induced by paracrine production of cytokines such as OSM from adjacent ASCs and that its ASC-derived overexpression indicates advanced proliferation, metastasis, and poor prognosis of breast carcinomas, possibly via RAGE activation.

In recent years, antiobesity drugs such as metformin have been widely studied in preclinical models and clinical trials for their effectiveness in improving clinical outcomes for patients with breast cancer [55]. Further studies are required to explore the potential application of anti-inflammatory drugs for obese patients to reduce the risk of breast cancer progression for S100A7^+^ patients. Therefore, not only the features of breast carcinoma themselves but also the status of stromal ASCs should be taken into account in the therapeutic application of ASC-derived stem cell use for breast reconstruction and the clinical management of patients with breast cancer.

## Additional files

Additional file 1: Supplementary information with materials and methods for supplementary figures, legends of Figures S1–S5, and Tables S1 and S2 listing primer and siRNA sequences used in this study. (DOCX 32 kb)

Additional file 2: Figure S1. Differentiation of adipocytes and its change of lipid droplet ratio by interaction with breast cancer cells, illustrating the 2D coculture and lipid droplet contents. (PDF 433 kb)

Additional file 3: Figure S2. Quantification of S100A7 mRNA expression in primary breast cancer tissues for comparison between normal epithelial cells and breast cancer cells. (PDF 161 kb)

Additional file 4: Figure S3. Detection of strong S100A7 expressions at the invasive front of MCF7 by interaction with ASCs, showing the Oil Red O and hematoxylin immunostaining of the cultured cells. (PDF 402 kb)

Additional file 5: Figure S4 Induction of oncostatin M in ASCs followed by coculture with breast cancer cells, showing the results of immunoblot assays. (PDF 181 kb)

Additional file 6: Figure S5. Web-based Kaplan-Meier analysis of S100A7 expression among patients with breast cancer, illustrating the prognosis of patients with breast cancer according to S100A7 expression using a public database. (PDF 163 kb)

## Abbreviations

3T3a: 3T3-L1 adipocyte stromal cell
ACM: Adipose stromal cell-derived conditioned medium
ASC: Adipose stromal cell
BCS: Breast cancer cancer-specific survival
BMI: Body mass index
C/EBPα: CCAAT/enhancer-binding protein α
CAA: Cancer-associated adipocyte
CAF: Cancer-associated fibroblast
CCM: Breast cancer cell-derived conditioned medium
CM: Conditioned medium
cRNA: Cyanine-3-labeled RNA
DEX: Dexamethasone
ER: Estrogen receptor
FABP4: Fatty acid-binding protein 4
hASC: Human adipocyte stromal cell
HER2: Human epidermal growth factor receptor 2
IBMX: 3-Isobutyl-1-methylxanthine
IF: Invasive front
IL: Interleukin
IT: Intratumoral
LI: Labeling index
mRNA: Messenger RNA
OSM: Oncostatin M
PBM-2: Preadipocyte basal medium 2
PCR: Polymerase chain reaction
PPARγ: Peroxisome proliferator-activated receptor-γ
PR: Progesterone receptor
Pre-3T3: 3T3-L1 preadipocyte
Pre-CM: Preadipocyte-derived conditioned medium
Pre-hASC: Human primary pre-adipocyte stromal cell
qRT-PCR: Quantitative real-time polymerase chain reaction
RAGE: Receptor for advanced glycation endproducts
RFS: Relapse-free survival
RT-PCR: Real-time polymerase chain reaction
siRNA: Small interfering RNA
STAT: Signal transducer and activator of transcription
