# Supplementary material for: Interaction with adipocyte stromal cells induces breast cancer malignancy via S100A7 upregulation in breast cancer microenvironment
Source: Breast Cancer Res. 2017 Jun 19;19:70. doi: 10.1186/s13058-017-0863-0 (PMC5477117; doi:10.1186/s13058-017-0863-0)
Supplement: Supplementary file 1 — Supplementary information with materials and methods for supplementary figures, legends of Figures S1–S5, and Tables S1 and S2 listing primer and siRNA sequences used in this study. (DOCX 32 kb) [file 13058_2017_863_MOESM1_ESM.docx]

**Supplementary Information**

**Materials and Methods for Supplementary Figures**

**Adipogenesis Assay**

The % lipid droplets were determined by oil red O staining and quantification of extracted dyes as described in manufacture’s protocol (Cayman Chemical Company, Michigan, USA). Briefly, adipose stromal cells (ASC) were fixed with 4-paraformaldehyde for 15 minutes, and rinsed twice with wash solution for five minutes. Filtered oil red o solution was then applied to the adipocytes for 20 minutes, followed by the several rinses with distilled water and wash solution in order to visualize lipid droplets under microscope without excess oild red staining. After the cells were completely dried, dye extraction solution was used to extract Oil Red O stains from the lipid droplets and its absorbance was measured for quantification as shown in Supplementary Figure S1B.

**Breast cancer tissue samples for mRNA quantification**

Snap-frozen samples of breast cancer mass and its surrounding normal epithelial tissues were collected for homogenization to extract RNA ( n =17). Mastectomy of these patients was performed between 2001 and 2003 at Tohoku University Hospital. Tumor cell infiltration in adjacent adipocyte tissues was confirmed in all the samples by hitological examination.

**2D Invasion Assay**

The 2D-invaion assay was assessed using 2-well silicon culture inserts (Ibidi, Munich, Germany), which was placed into 12-well plate. 3T3-L1 preadipocytes were seeded and differentiated into mature adipocytes in one side of the well. Then, MCF7 was seeded on the left well 70µl at 1x10^ 5 ml and incubated for 24 h. The culture inserts were then removed and 50mm gap was left between 3T3a and MCF7. After 24-h incubation, cells were fixed with 4% paraformaldehyde, and the cells were stained by Oil Red O, Hematoxylin, and DAB for lipids, nuclear, and S100A7 immunoreactivity, respectively. The primary antibody for S100A7 was used in the same condition as immunohistochemistry.

**Kaplan-Meier survival curve based on publically available data**

To determine S100A7 immunoreactivity and its relationship with breast cancer clinical outcome, we used web-based Kaplan−Meier analysis using Kaplan−Meier Plotter (http://kmplot.com/analysis/index.php?p=service&cancer=breast) for 3455 breast cancer patients, downloaded from GEO (Affymetrix HGU133A and HGU133+2 microarrays) ([1](#_ENREF_1)).

**Legends of Supplementary Figures**

**Figure S1. Differentiation of adipocytes and its change of lipid droplets ratio by interaction with breast cancer cells**

**A.** 3T3-L1 preadipocytes were differentiated and lipids were stained by Oil Red O stain, and nuclear was counter-stained with hematoxylin. **B.** Accumulation of lipid droplets was measured by extraction of Oil Red O staining at different time points. **C.** 2D co-culture system using breast cancer cells and preadiopcytes/ adipose stromal cells (ASC), **D.** Decreased lipid droplet ratio of ASC by treatment of each breast cancer cell derived conditioned medium.

**Figure S2. Quantification of S100A7 expression in primary breast cancer tissues**

S100A7 mRNA was examined among 17 breast cancer patients with with tumor infiltration in surrounding adipose tissues to compare the expression levels between its breast cancer cells and normal epithelial cells. N = normal, T = tumor

**Figure S3. Detection of strong S100A7 expressions at the invasive front of MCF7 by interaction with ASC**

**A.** Schematic illustration indicates the areas of the following figures by dotted boxes. Adipocytes (Ad), MCF7(Ca). Arrows indicate S100A7 expression in MCF7 followed by simulation of adipocytes, **B.** at the invasive front of adipocytes and **C.** edge of cancer mass with morphological alteration. **D.** Lipid droplets embedded in MCF7 were detected followed by carcinoma-ASC interaction.

**Figure S4. Induction of Oncostatin-M in ASC followed by co-culture with breast cancer cells**

Immunoblot analysis shows increased oncostation-M (OSM) expressions in 3T3-L1 adipocytes (3T3a) by interaction with MCF7, T47D, and ZR-75-1, compared to mono-culture (ctrl).

**Figure S5. Web-based Kaplan−Meier analysis of S100A7 expression among breast cancer patients**

**A***.* Recurrence-free survival (RFS) rate (n = 3455) and overall survival (OS) rate (n = 1115) for all types of breast cancer patients in publically available data. **B***.* RFS rate (n = 788) and OS rate (n = 292) for ER− breast cancer patients. *P* values were determined by the log-rank test.

**Supplementary Table 1.** Primer Sequences used in this study

**Human Primers**

| **NCBI ID** | **Gene** | **Upper 5'-3'** | **Lower 3'-5'** |
| --- | --- | --- | --- |
| NM_001101.3 | β-actin | CACTGTGTTGGCGTACAGGT | TCATCACCATTGGCAATGAG |
| NM_002963.3 | S100A7 | AAATACACCAGACGTGATGACA | GACATCGGCGAGGTAATTTG |
| NM_015869 | PPARγ | GTTGACTTCTCCAGCATTTCTA | TCCACTTTGATTGCACTTTG |
| NM_004364 | C/EBPα | CGAGCCAGGACTAGGAGATTC | CCTCATCTTAGACGCACCA |
| NM_001442.2 | FABP4 | GCCAGGAATTTGACGAAGT | CTCATAAACTCTCGTGGAAGTG |
| NM_000565 | IL6R | TGTGCGTCGCCAGTAGTGT | CACGGCAGTGACTGTGATG |
| NM_002184 | gp130 | GCCCTTGGGAAGGTTACATC | AATACTTGGGTTGGTCCATGTC |
| NM_000600 | IL6 | CTTCCAATCTGGATTCAATG | CAAATCTGTTCTGGAGGTACTCTA |
| NM_002309 | LIF | CCGCATAGTCGTGTACCTTG | CGTTGAGCTTGCTGTGGA |
| NM_006837.2 | Jab1 | CCATTTGTAGCAGTGGTGATTG | CAGGAGGTTTGTAGCCCTTTG |
| AB036432.1 | RAGE | GAAGCCCCTGGTGCCTAAT | GCAGTGTGAAGAGCCCTGTC |
|  |  |  |  |
| **Mouse Primers** | |  |  |
| **NCBI ID** | **Gene** | **Upper 5'-3'** | **Lower 3'-5'** |
| NM_007393.3 | β-actin | CCTAGCACCATGAAGATCAAGA | GGTGTAAAACGCAGCTCAG |
| NM_011146 | Pparγ | TCAGAAGTGCCTTGCTGTG | TCAGCAGACTCTGGGTTCA |
| NM_007678 | C/ebpα | GAAGTCGGTGGACAAGAACA | CAGGCGGTCATTGTCACT |
| NM_024406 | Fabp4 | TGTGATGCCTTTGTGGGA | TGACCGGATGGTGACCAA |
|  |  |  |  |

**Supplementary Table 2.** Small interference RNAs used in this study

| **Name** | | | **Sense 5'-3'** | | **Anti-sense 3'-5'** |
| --- | --- | --- | --- | --- | --- |
| S100A7 #1 | | CAGACGUGAUGACAAGAUUTT | | AAUCUUGUCAUCACGUCUGTT | |
| S100A7 #2 | | CAAAUUCCACCUCGCCGAUGUTT | | ACAUCGGCGAGGUAAUUUGTT | |
|  |  |  | |  | |

**Reference**

1. Gyorffy B, Lanczky A, Eklund AC, Denkert C, Budczies J, Li Q, et al. An online survival analysis tool to rapidly assess the effect of 22,277 genes on breast cancer prognosis using microarray data of 1,809 patients. Breast Cancer Res Treat 2010;123(3):725-31.
