# Supplementary figures and images for: Interaction with adipocyte stromal cells induces breast cancer malignancy via S100A7 upregulation in breast cancer microenvironment
Source: Breast Cancer Res. 2017 Jun 19;19:70. doi: 10.1186/s13058-017-0863-0 (PMC5477117; doi:10.1186/s13058-017-0863-0)

Supplementary Figure 1.

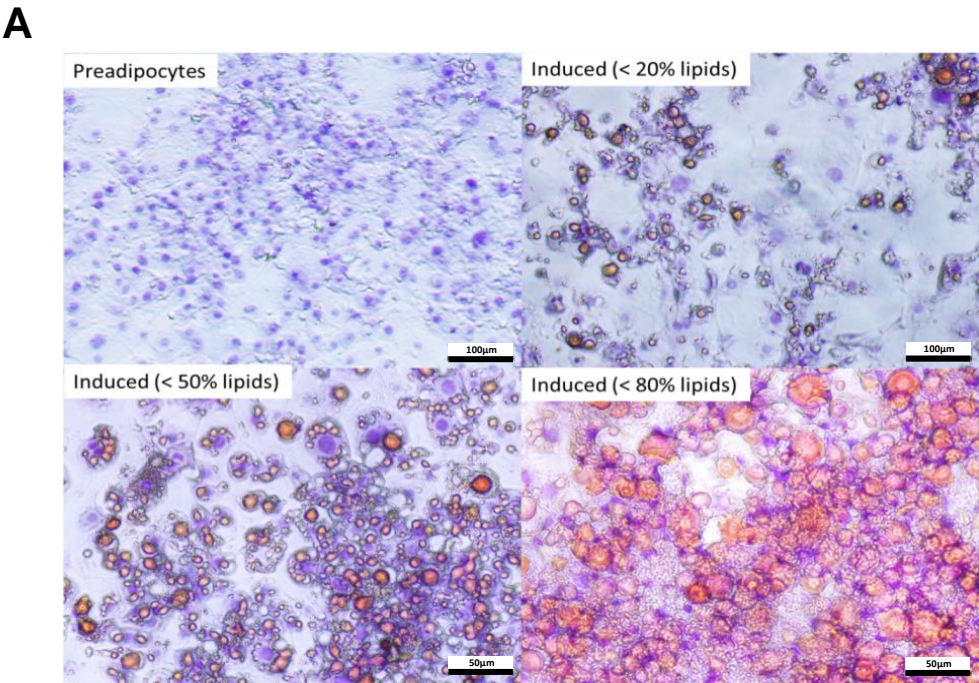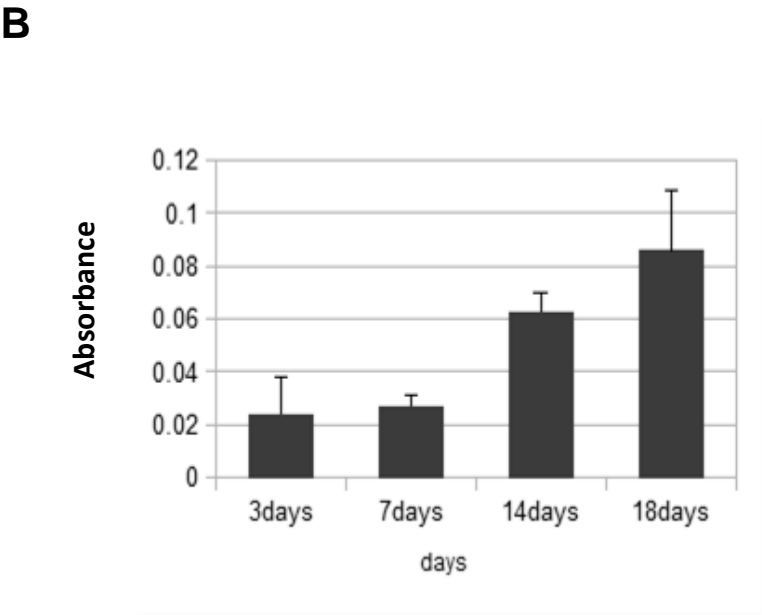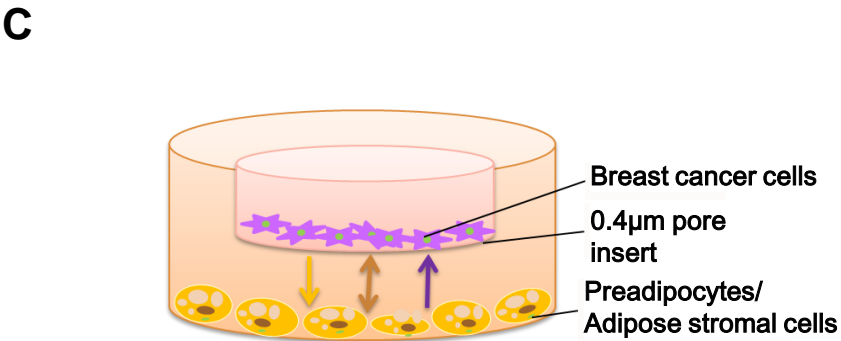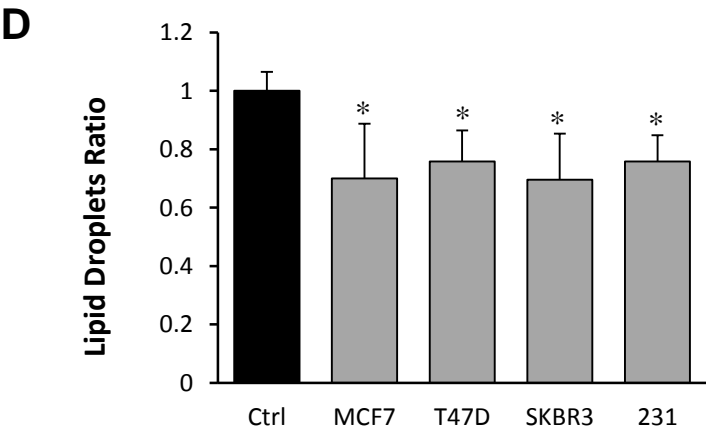

Supplement: Supplementary file 2 — Differentiation of adipocytes and its change of lipid droplet ratio by interaction with breast cancer cells, illustrating the 2D coculture and lipid droplet contents. (PDF 433 kb) [file 13058_2017_863_MOESM2_ESM.pdf]

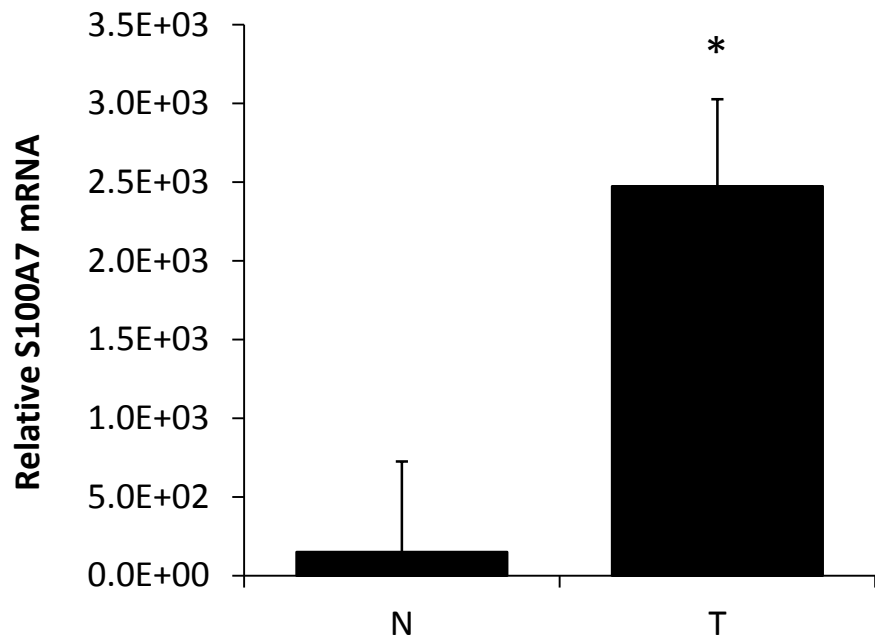

Supplement: Supplementary file 3 — Quantification of S100A7 mRNA expression in primary breast cancer tissues for comparison between normal epithelial cells and breast cancer cells. (PDF 161 kb) [file 13058_2017_863_MOESM3_ESM.pdf]

Supplementary Figure 3.

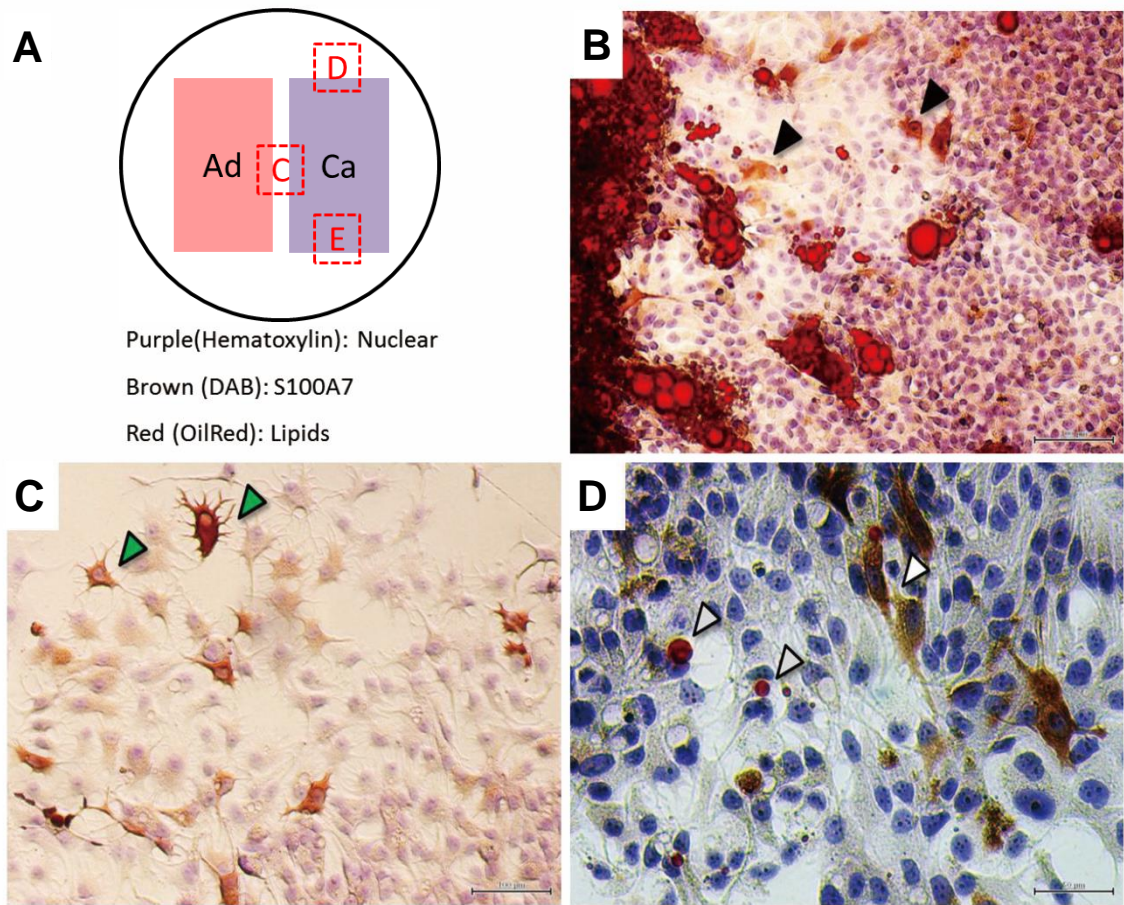

Supplement: Supplementary file 4 — Detection of strong S100A7 expressions at the invasive front of MCF7 by interaction with ASCs, showing the Oil Red O and hematoxylin immunostaining of the cultured cells. (PDF 402 kb) [file 13058_2017_863_MOESM4_ESM.pdf]

Supplementary Figure 4.

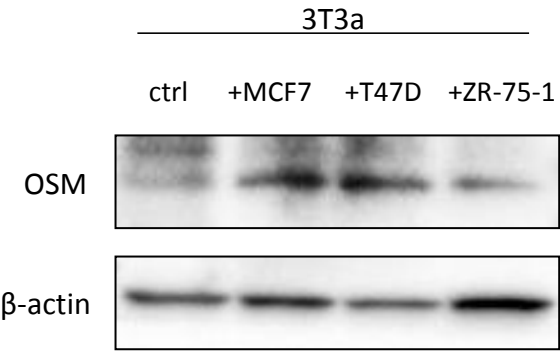

Supplement: Supplementary file 5 — Induction of oncostatin M in ASCs followed by coculture with breast cancer cells, showing the results of immunoblot assays. (PDF 181 kb) [file 13058_2017_863_MOESM5_ESM.pdf]

**A**

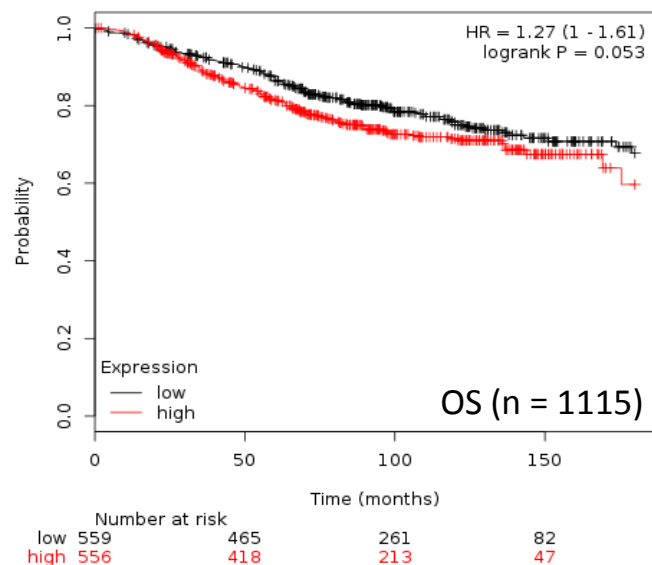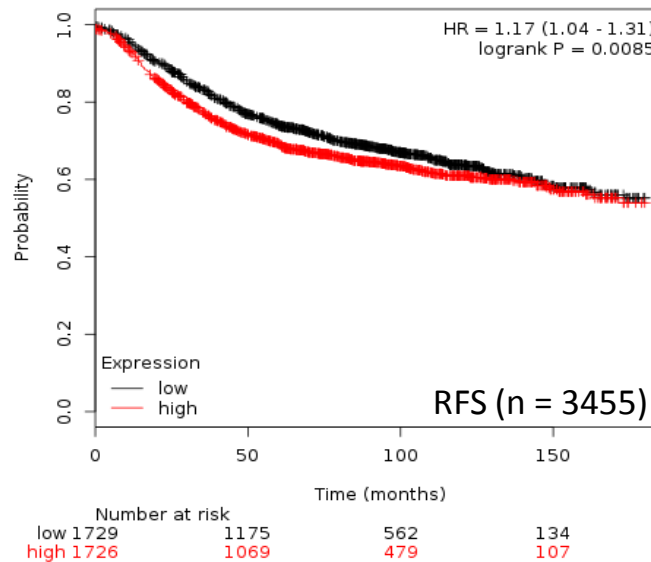

**B**

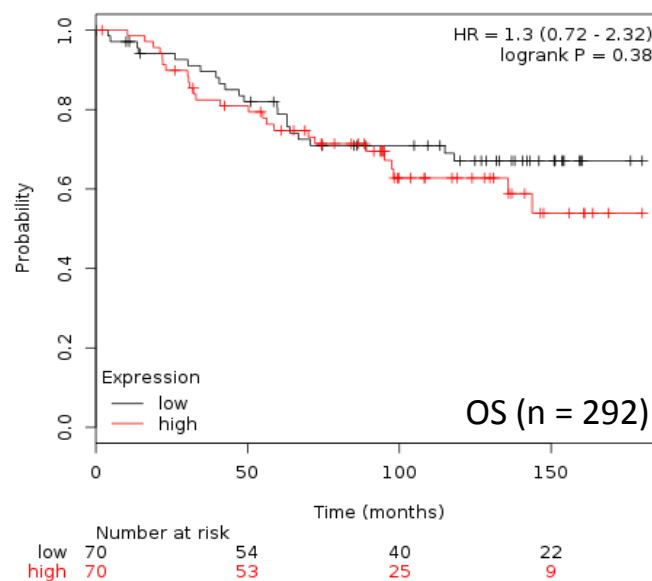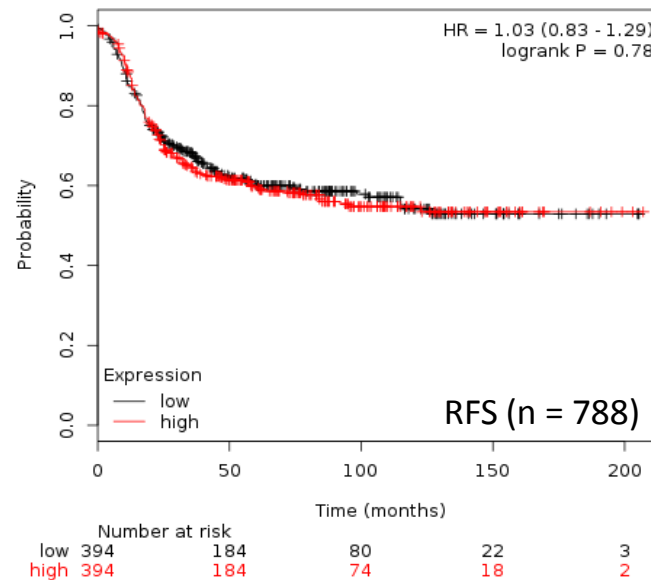

Supplement: Supplementary file 6 — Web-based Kaplan-Meier analysis of S100A7 expression among patients with breast cancer, illustrating the prognosis of patients with breast cancer according to S100A7 expression using a public database. (PDF 163 kb) [file 13058_2017_863_MOESM6_ESM.pdf]
